# Sequencing of the Canine Cytochrome P450 CYP2C41 Gene and Genotyping of Its Polymorphic Occurrence in 36 Dog Breeds

**DOI:** 10.3389/fvets.2021.663175

**Published:** 2021-04-22

**Authors:** Emre Karakus, Clarissa Prinzinger, Silke Leiting, Joachim Geyer

**Affiliations:** Faculty of Veterinary Medicine, Institute of Pharmacology and Toxicology, Justus Liebig University Giessen, Giessen, Germany

**Keywords:** CYP2C41, dog, drug metabolism, pharmacogenetic, genotyping, gene deletion

## Abstract

Cytochrome P450 (CYP) drug metabolizing enzymes play an important role in efficient drug metabolism and elimination. Many CYPs are polymorphic and, thereby, drug metabolism can vary between individuals. In the case of canine CYP2C41, gene polymorphism was identified. However, as the first available canine genome sequences all were CYP2C41 negative, this polymorphism could not be clarified at the genomic level. The present study provides an exact characterization of the CYP2C41 gene deletion polymorphism at the genomic level and presents a PCR-based genotyping method that was used for CYP2C41 genotyping of 1,089 individual subjects from 36 different dog breeds. None of the Bearded Collie, Bernese Mountain, Boxer, Briard, French Bulldog or Irish Wolfhound subjects had the CYP2C41 gene in their genomes. In contrast, in the Chinese Char-Pei, Siberian Husky, Schapendoes and Kangal breeds, the CYP2C41 allele frequency was very high, with values of 67, 57, 43, and 34%, respectively. Interestingly, the site of gene deletion was identical for all CYP2C41 negative dogs, and all CYP2C41 positive dogs showed highly homologous sequence domains upstream and downstream from the CYP2C41 gene. CYP2C41 genotyping can now be routinely used in future pharmacokinetic studies in canines, in order to identify genetically-based poor or extensive drug metabolizers. This, together with more extensive *in vitro* drug screening for CYP2C41 substrates will help to determine the clinical relevance of CYP2C41, and to optimize drug treatment. Although the relative abundance of the CYP2C41 protein in the canine liver seems to not be very high, this CYP could substantially contribute to hepatic drug metabolism in dogs expressing CYP2C41 from both alleles and, when CYP2C41 shows higher catalytic activity to a given drug than other hepatic metabolic enzymes.

## Introduction

Cytochrome P450 (CYP) metabolizing enzymes play an important role in the efficient metabolism and elimination of many drugs (1, 2). It is well-recognized that the activity of CYP-mediated drug metabolism varies considerably between species, breeds and individuals, which is often based on genetic variability (3). As a consequence, clinical outcomes after standard dosing can be less efficient due to high CYP catalytic activity, high drug clearance, and, as a result, suboptimal plasma levels (4). Individuals with this phenotype often are referred to as extensive metabolizers. Conversely, CYP deficiency or reduced CYP catalytic activity can result in lower drug clearance, higher drug plasma levels, and, as a result, increases in adverse drug reactions and drug toxicities (5). As an example, the premature stop codon mutation c.1117C>T (R373X) in canine CYP1A2 results in a complete loss of the hepatic CYP1A2 protein, and a reduction in the associated catalytic activity in homozygous dogs (6, 7). This single nucleotide polymorphism (SNP) was discovered during preclinical testing of novel drug candidates (AC-3933 and YM-64227) and was associated with significantly increased drug serum levels (8–10). Genetic variation can also affect the translation efficiency of CYP-coding mRNAs, with the consequence of lower drug metabolism, which is the case for the CYP2B11 haplotypes H2 and H3 (11, 12). Larger chromosomal regions can also be polymorphic, as is the case for the sparsely characterized CYP2C41. Blaisdell et al. found in 1998 that CYP2C41 cDNA can only be cloned from one out of nine Beagle dogs, whereas CYP2C21 cDNA was present in all nine subjects (13). However, in contrast to the other canine CYPs, the CYP2C41 gene was not present in the first available dog genomes and, therefore, this polymorphism could not be analyzed at the genomic level. Therefore, the goal of the present study was to clone the full sequence of the canine CYP2C41 gene, in order to provide the basis for the development of appropriate genotyping methods. In the present study a PCR-based method for CYP2C41 genotyping is presented as well as data for the breed distribution of this gene deletion polymorphism from 1,089 individual subjects from 36 different dog breeds.

## Materials and Methods

### DNA Samples

Blood samples from client-owned dogs were originally nt230(del4) MDR1 genotyped for clinical diagnostic reasons as part of the pharmacogenetic diagnostic service at the host institute as previously reported (14). Briefly, genomic DNA was isolated from 200 μl of EDTA-anticoagulated blood using a DNA isolation kit following manufacturer's instructions (NucleoSpin Blood QuickPure, Macherey, Düren, Germany). Some of these DNA samples then were used for retrospective CYP2C41 genotyping. The dog owners gave consent that the DNA samples can be used for subsequent scientific studies instead of being discarded. No samples were taken specifically for the retrospective analysis presented in the current manuscript and, therefore, this re-analysis did not require ethical approval. In total, 1,089 samples from 36 dog breeds were re-analyzed.

### Full-Length PCR-Amplification and Sequencing of the Canine CYP2C41 Gene

DNA from a CYP2C41-positive Shetland Sheepdog was used to amplify the full-length CYP2C41 gene. Based on sequence alignments between the CYP2C21 (GenBank accession number NM_001197044) and CYP2C41 (GenBank accession number NM_001003334) cDNAs, the exon structure of the CYP2C41 gene was predicted. Respective exon-spanning forward and reverse primers were designed (see Table 1) and used for the amplification of exons 1–9 in both genes. The amplification of all exons was performed in a peqSTAR thermocycler (PeqLab, Erlangen, Germany) with a total volume of 50 μl. The reactions contained 10 μl 5X Phusion HF Buffer, 1 μl 10 mM dNTPs, 0.5 μM of the respective forward and reverse primer, 0.5 μl Phusion High-Fidelity DNA Polymerase (ThermoFisher Scientific, Dreieich, Germany), and 5 μl of DNA. The PCR cycling parameters were as follows: initial denaturation at 98°C for 30 s, 30 cycles of 98°C for 10 s, 52–57°C (see Table 1) for 20 s, 72°C for 20 s, and final elongation at 72°C for 7 min. All PCR products were separated by electrophoresis in 2.5% agarose gels and stained with ethidium bromide (Sigma Aldrich, Taufkirchen, Germany). The relevant amplicons were excised under UV light, extracted with the GeneJET gel extraction kit (ThermoFisher Scientific), and subjected to DNA sequencing. DNA sequencing was done by Microsynth SeqLab (Göttingen, Germany) using Sanger sequencing. The recommended concentrations for the PCR products were 1.5 ng/μl per 100 bp in ddH_2_O. Sequencing primers (20 μM) and DNA template solutions were sent separately in 1.5 ml reaction cups. In a similar manner, primers were used to amplify the intron sequences. Sequence alignments were performed with the MegAlign tool of the DNASTAR Lasergene software. All PCR fragments were assembled and the full-length genomic CYP2C41 sequence was deposited into the GenBank database with accession number HF677515.

**Table 1:** Primers used for PCR.

**Primer name CYP2C21 (sequence 5**^**′**^ → 3^**′**^**)**		**Annealing temp. (^**°**^C)**	**Product size (bp)**	**Primer name CYP2C41 (sequence 5**^**′**^ → 3^**′**^**)**		**Annealing temp. (^**°**^C)**	**Product size (bp)**
EX1-F	TGG ATC TCT TCA TAG TTC TGG	53	163	EX1-F	CAT GGA TCC AGT TGT GGT TC	52	148
EX1-R	CTT AGG GAT TTG CTG ACA TTC			EX1-R	GAT GTC CTT ATC TAA CTG TAG G		
EX2-F	TTA TGG CCC TGT GTT CAC TG	53	128	EX2-F	CTA TGG CCC TGT ATT CAC TC	54	147
EX2-R	ACA ATG GGA AAT GGC CTC TG			EX2-R	CCT CCA CTA ACT TTT TCG GC		
EX3-F	AAA TGG AAG CAA ACC CGG C	53	110	EX3-F	CAG TGG AAA CAG ATG GAA GG	52	126
EX3-R	GCT TCC ACT AGA TAC AAG GC			EX3-R	CTC AAC TCT TCT ACA AGG TAG		
EX4-F	TCC CTG TGA TCC TAC TTT C	53	150	EX4-F	TAC CAT GTG ATC CCA CTT TTG	52	153
EX4-R	CAG GAG GTG CTT GAA ATT AG			EX4-R	TCC ATG GGG AGC TCA AAA TC		
EX5-F	CTC TAC AAT GCT TTC CCT C	53	157	EX5-F	TAC AAT AGT TTC CCT GCT CTC	57	149
EX5-R	AGT AGT CAA TAA AGT CCC GAG			EX5-R	TCA ATG AAA TCC CGA GGA TTG		
EX6-F	CAG TCT GAA TTT ACC ATG GAC	53	103	EX6-F	AAA GCA CAA CCA GCC ATT GG	52	140
EX6-R	GCA CCA ATA GTC CGT ATC TC			EX6-R	CTG TGA CTT CTG GAT GTT TC		
EX7-F	TCA TCG TGT AGT TGG CAG AC	53	152	EX7-F	TCC AGG AAG AGA TTG ACC	52	182
EX7-R	CTC TAA ACT TGA TGT CCT GAG			EX7-R	CTT GGG GAT GAC ATA GTT TC		
EX8-F	CAT CTC TGA CTT CTG TCC TG	53	123	EX8-F	TGT CTT CTG TGC TAT CTG ATG	54	115
EX8–R	CTG AGA AGG CCA TGA AGT AG			EX8-R	GAG AAA GCC ATG AAG TAG TC		
EX9-F	AGA GAG TTT GTG TTG GAG AAG	53	155	EX9-F	ACG AAT TTG TGT GGG AGA AG	54	246
EX9-R	ATA GGA AGG TGG TGT AGC AC			EX9-R	GAA TGA TAC CCC AGA GGA AGA G		
**Primer name**	** Primer sequence (5′ → 3′)**				**Annealing temp. (°C)**	**Product size (bp)**	
SOR-F3	GAA TTC GGT CAT TAG ACA AGT TGA AAA AC				59	506	
SOR-R3	CTG GTG AGT TCA GAG TTT GCT						
SOR-F3	GAA TTC GGT CAT TAG ACA AGT TGA AAA AC				59	366	
SOR-R4	GAG GAG ACA CTT TGA AAC TAT GTA ACT ATT C						
SOR-F4	CTT TGG GCC AGT GTG ACC				69	1018	
SOR-R3	CTG GTG AGT TCA GAG TTT GCT						

### Amplification and Sequencing of the Sites of Recombination Flanking the CYP2C41 Gene

To amplify and sequence the sites of recombination (SOR), dog DNA samples were prescreened by PCR for the presence of the CYP2C41 gene (primers EX7-F/R, CYP2C41 exon 7) and the CYP2C21 gene, which was used as a control (primers EX3-F/R, CYP2C21 exon 3) (see Table 1). Then, the sites of recombination of selected CYP2C41-positive dogs were amplified with the primers SOR-F3/R4 (break point SOR1) and SOR-F4/R3 (break point SOR2). In CYP2C41 negative dogs, the SOR break point was amplified with the primers SOR-F3/R3. All PCR reactions were performed using a peqSTAR thermocycler instrument with a total volume of 10 μl, containing 1 μl of genomic DNA, 1 μM of each primer, 1 mM dNTPs mix, 1 μl of 10 × PCR buffer, and 0.5 units DreamTaq DNA Polymerase (ThermoFisher Scientific). The PCR conditions were as follows: initial denaturation at 95°C for 3 min, 30 cycles of 95°C for 30 s, 59°C/69°C (see Table 1) for 30 s, 72°C for 60 s, and final elongation at 72°C for 5 min. All PCR products were analyzed by electrophoresis in 2 % agarose gels and gel extraction and sequencing procedures were performed as mentioned above.

### Screening for the CYP2C41 Gene by Real-Time PCR and Melting Curve Analysis

Presence of the CYP2C41 gene was screened by multiplex real-time PCR on a StepOnePlus Real-Time PCR instrument (Applied Biosystem, Darmstadt, Germany), using the fluorescent dye SYBR Green PCR-Master-Mix (PowerUp SYBR Master Mix A25742, ThermoFisher Scientific). PCR mixtures contained 0.1 μM EX7-F/R primers for CYP2C41, and 0.3 μM EX3-F/R primers for CYP2C21, 5 μl SYBR green master mix, and 1 μl of DNA in a final volume of 10 μl. General PCR conditions were initial denaturation at 95°C for 10 min and 40 cycles of 95°C for 15 s, 56°C for 30 s, and 72°C for 30 s. The level of fluorescence of the dsDNA-bound SYBR green dye was recorded for each sample after product extension, which allowed close monitoring of the amplification reaction. Since each amplified fragment is characterized by its apparent melting temperature (Tm), which is a function of product length and base composition, a subsequent melting curve analysis could be performed under the following conditions: 95°C for 15 s, 60°C for 1 min, and then increasing to 95°C at five acquisitions per °C. Each amplified fragment was identified by converting its specific melting curve, measured as fluorescence emission decrease, to a melting peak by the software.

### CYP1A2 and MDR1 Genotyping With TaqMan Allelic Discrimination Analysis

Fluorogenic 5′ nuclease TaqMan allelic discrimination (AD) analysis was used for CYP1A2 1117C>T and nt230(del4) MDR1 genotyping, as previously reported (15, 16) using gene-specific oligonucleotide primers and fluorescence-labeled allele-specific oligonucleotide probes (Applied Biosystems). Real-time PCR amplification was carried out in a total reaction volume of 25 μl, consisting of 12.5 μl TaqMan genotyping master mix (Applied Biosystems), including AmpliTaq Gold DNA polymerase, dNTP mix, reaction buffer and ROX reference dye, 2.5 μl TaqMan AD assay, and 100 ng of genomic DNA. The samples were amplified in 96-well optical plates on a StepOnePlus real-time PCR instrument (Applied Biosystem). The amplification reaction was started with activation of the AmpliTaq Gold DNA polymerase at 95°C for 10 min, before 40 cycles of 92°C × 15 s and 60°C × 30 s were applied.

## Results

### Polymorphic Occurrence of the Entire CYP2C41 Gene in Dogs

**Figure 1:**
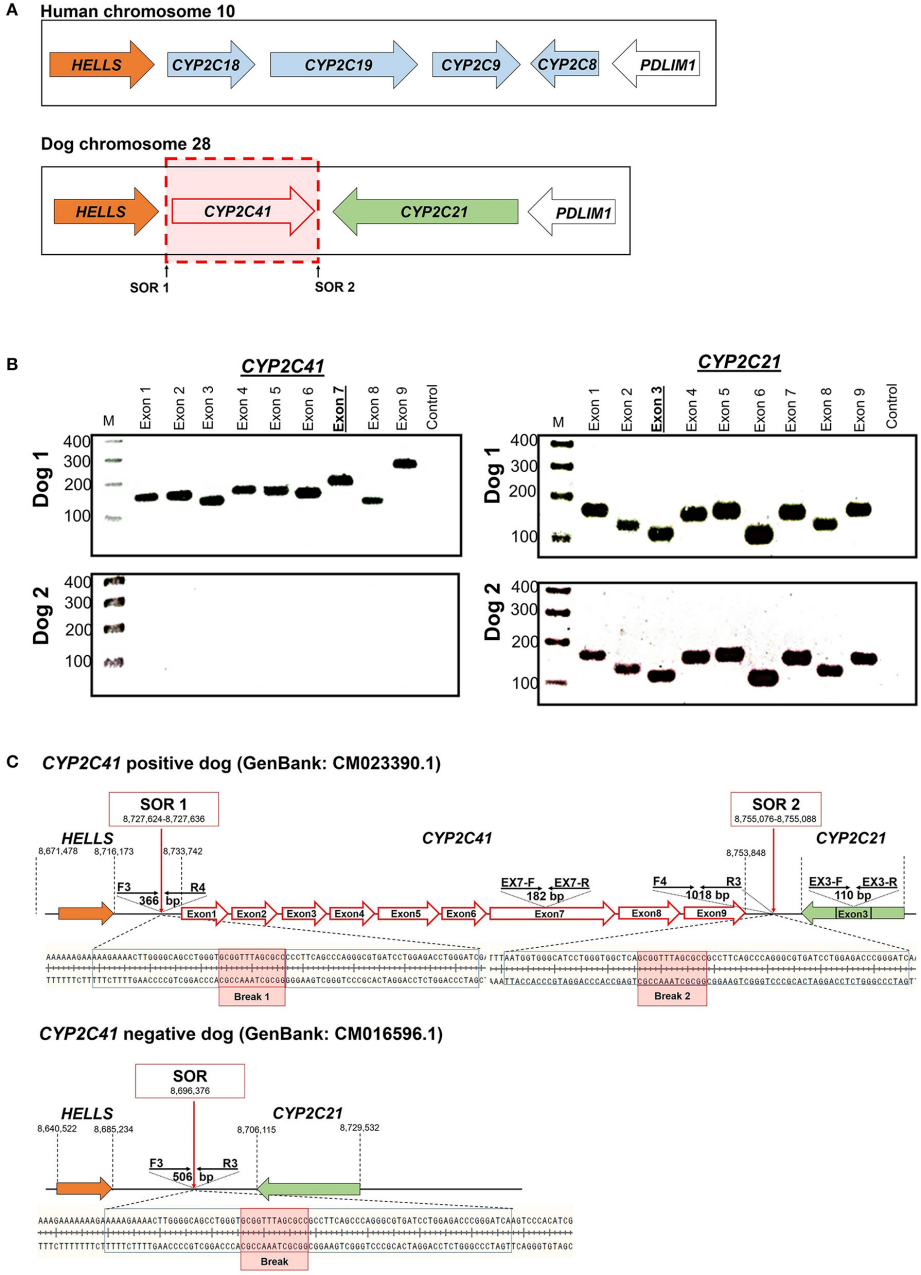
Genomic CYP2C41 gene deletion polymorphism. **(A)** Chromosomal localization and organization of human and canine CYP2C gene clusters on chromosomes 10 and 28, respectively. Gene arrangements with corresponding proportions derived from the NCBI Genome Data Viewer (www.ncbi.nlm.nih.gov). Both gene clusters are bordered by the HELLS and PDLIM1 genes. The canine CYP2C41 gene was schematically introduced based on experimental data from the present study, which demonstrated localization downstream of the HELLS gene. Sites of recombination (SOR 1 and SOR 2) are indicated. **(B)** Oligonucleotide primers specific for each proposed exon of the CYP2C41 gene were used for PCR amplification from DNAs from different subjects. Data are representatively shown for a CYP2C41 positive subject (dog 1) and a CYP2C41 negative subject (dog 2). As a control, all CYP2C21 gene coding exons were also amplified with gene-specific primers. All amplicons were separated by agarose gel electrophoresis. M, molecular weight DNA marker (100-400 bp). The underlined exons were also examined in subsequent analyses. **(C)** Schematic presentation of the CYP2C41 genotyping strategy. Subjects were screened for the presence of the CYP2C41 gene with the oligonucleotide primers EX7-F/R, both located on exon 7 of the CYP2C41 gene. The primers EX3-F/R were used as controls to amplify exon 3 from the CYP2C21 gene. Amplification with the primers SOR-F3/R4 or SOR-F4/R3 only produced positive results in the presence of the CYP2C41 gene, whereas amplicons with the primers SOR-F3/R3 indicated the CYP2C41 gene deletion. Of note, DNA sequence patterns were identical at break points 1 and 2 of all CYP2C41 positive subjects (indicated by red boxes). In addition, the exact positions of the respective gene segments are indicated.

When this project was started in 2012, canine genetic information was still limited, and the only available genome sequence was from a CYP2C41 negative dog (Boxer named Tasha, see Table 2). Therefore, the presence and chromosomal localization of the CYP2C41 gene was not clear at that time. Direct comparison between human chromosome 10 and dog chromosome 28 segments revealed similar CYP2C gene regions, flanked by the genes HELLS and PDLIM1 in both species (Figure 1A). As all four human CYP2C genes (CYP2C8, C9, C18, and C19) typically cluster, localization of the canine CYP2C41 gene was predicted to be in proximity with the CYP2C21 gene. Based on data from the present study, the CYP2C41 gene could be clearly localized between the HELLS and CYP2C21 genes in the same chromosomal orientation as the human CYP2C18, C19, and C9 genes.

**Table 2:** Available genomic CYP2C41 sequences of the dog.

**Dog breed**	**Genomic DNA/cDNA**	**GenBank (assembly) accession number**	**CYP2C41 gene status**	**Available since/current version (Reference)**
Not specified	cDNA	NM_001003334.1 AF016248.1	Full-length CYP2C41 cDNA	1998 (13)
Mixed breed	cDNA	HF679527.1	Full-length CYP2C41 cDNA	2013 (Prinzinger and Geyer)
Shetland Sheepdog	Genomic DNA	HF677515.1	Full-length CYP2C41 gene	2013 (Prinzinger and Geyer)
Basenji	Genome assembly Basenji_breed-1.1	GCA_004886185.1	CYP2C41 negative	2019
Great Dane	Genome assembly UMICH_Zoey_3.1	GCA_005444595.1 NC_049288.1	CYP2C41 negative	2019
Labrador Retriever	Genomic DNA chromosome 28b	GCA_008641245.1 CP050626	CYP2C41 positive	2020
German Shepherd	Genomic DNA chromosome 28	CM021989	CYP2C41 negative	2020
Boxer	Genome assembly Dog10K_Boxer_Tasha	NC_006610.4	CYP2C41 negative	2021
Basenji	Genome assembly UNSW_CanFamBas_1.0	NC_049769.1	CYP2C41 positive	2021

Originally it was not known if the entire CYP2C41 gene was deficient in canine breeds or just parts of it. Therefore, the previously published 1,470 bp CYP2C41 cDNA (13) was used for sequence alignments in sections of the genomic sequence of the canine CYP2C21 gene to predict potential exon regions and exon/exon boundaries of CYP2C41. Then, based on CYP2C41 cDNA, specific oligonucleotide forward and reverse primers were designed for each predicted exon, and used for PCR amplification from different canine genomic DNAs. For comparison, primers specific for each CYP2C21 exon were used. As indicated in Figure 1B for two representative subjects, all nine predicted CYP2C41 exons could be amplified with the predicted amplicon lengths (dog 1), or all CYP2C41 PCR reactions were negative (dog 2). This indicates polymorphic absence of the entire CYP2C41 gene and not only parts of it. For comparison, all nine CYP2C21 exons from both subjects were amplified, demonstrating presence of the full-length CYP2C21 gene independent of the presence of the CYP2C41 gene.

### Full-Length CYP2C41 Gene Sequencing and Assembly

In the next step, the predicted exon sequences were used for intron-spanning PCR amplification with different pairs of forward and reverse primers from genomic DNA of CYP2C41 positive subjects. All amplicons were subjected to DNA sequencing. Primer sequences were designed in such a way as to obtain overlapping DNA fragments, so that the full-length genomic sequence of the canine CYP2C41 gene could be assembled. This genomic sequence was entered into the GenBank database in 2013 under accession number HF677515 (see Table 2). Comparative analysis of the exon-intron organization of the CYP2C41 and CYP2C21 genes revealed identical exon sizes for both genes (see Tables 3, 4, respectively), but overall, the CYP2C21 gene is somewhat larger compared to the CYP2C41 gene. However, at the time this procedure was carried out, the exact chromosomal location of the CYP2C41 gene was still unknown and it was not possible, despite intensive efforts using long-range PCR with the flanking HELLS and PDLIM1 genes, to specify the localization of the newly sequenced CYP2C41 gene within the CYP2C gene cluster. During revision of this manuscript, the gray wolf (*Canis lupus*) genome assembly of chromosome 28 was made available at GenBank with accession number HG994413. This sequence was used for direct comparison of the CYP2C gene cluster between the dog and the wolf. Interestingly, the gray wolf exhibits two genes, CYP2C21 and CYP2C41, which are highly similar to the respective canine orthologs.

**Table 3:** Exon-intron organization of the canine CYP2C41 gene.

**Exon**	**CDS length (bp)**	**5^**′**^-Splice donor**	**3^**′**^-Splice acceptor**	**Intron size (bp)**
		**ATG**GAT.		
1	165	AGTAAT/gtaagt	tctcag/CTCTCA	1,411
2	163	GACACG/gtaggt	tggtag/GAATCA	169
3	150	CCAATG/gtgagt	ctttag/CCTTAC	4,249
4	161	ATCCAG/gtgagg	tttaag/GCCTAC	2,452
5	177	GAACAG/gtaaaa	tcctag/GAAAAG	2,859
6	142	TCACAG/gtatgg	tatcag/TGAAAG	159
7	188	CCCAAG/gtaaga	ttccag/GGCACA	5,452
8	142	CAGCAG/gtaata	tttcag/GAAAAC	1,820
9	182	GTG**TGA**		

*Nucleotide sequences of the exon/intron junctions are indicated according to GenBank accession numbers HF677515, NC_049769.1, and GCA_008641245.1. Exon sequences are shown in uppercase letters, intron sequences are in lowercase. Start codon and stop codon are indicated in bold*.

**Table 4:** Exon-intron organization of the canine CYP2C21 gene.

**Exon**	**CDS length (bp)**	**5^**′**^-Splice donor**	**3^**′**^-Splice acceptor**	**Intron size (bp)**
		**ATG**ACT.		
1	339	AGCAAG/gtaggt	tcccag/CTAGCA	1,901
2	163	GATTAG/gtatgt	tgatag/GAATTG	1,719
3	150	CCAACG/gtgtgt	ttttag/CATCTC	2,137
4	161	ATACAG/gtaagg	tttcag/CTCTAC	1,804
5	177	GAAAAG/gtaaaa	ttgtag/GAAAAA	950
6	142	TCACAG/gtatgg	tgccag/CTAAAG	2,653
7	188	CCCAAG/gtgaga	tatcag/GGCACA	3,935
8	142	CAGCAG/gcaagc	ttttag/GGAAGA	6,360
9	182	GTC**TGA**		

*Nucleotide sequences of the exon/intron junctions are indicated. Exon sequences are shown in uppercase letters, intron sequences are in lowercase. Start codon and stop codon are indicated in bold*.

### Screening for CYP2C41 Positive Canine Subjects by Multiplex Real-Time PCR

**Figure 2:**
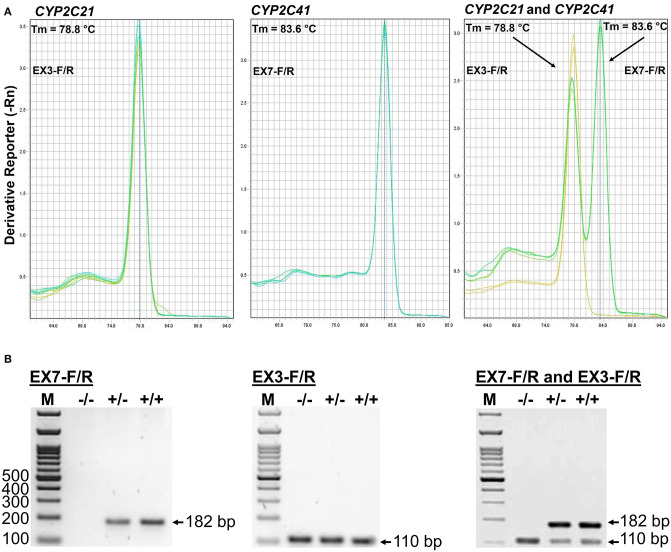
Screening for presence of the CYP2C41 gene. **(A)** Multiplex PCR and melting curve analysis for simultaneous detection of the CYP2C21 (primers EX3-F/R, Tm = 78.8°C) and CYP2C41 (primers EX7-F/R, Tm = 83.6°C) genes. Multiplex PCR with primers EX3-F/R and EX7-F/R revealed identical melting curves as the single gene PCRs with primers EX3-F/R or EX7-F/R. **(B)** The PCR products were separated on 2% agarose gels followed by visualization with ethidium. Representative data for all three CYP2C41 genotypes: (+/+), homozygous positive; (+/–), heterozygous; and (–/–), homozygous negative. Amplification with the primers EX7-F/R revealed PCR fragments of 182 bp, indicating the presence of the CYP2C41 gene, and amplification of CYP2C21 exon 3 with the primers EX3-F/R revealed 110 bp fragments. By using multiplex PCR, both fragments were detected. M, molecular weight DNA marker.

The CYP2C41 genomic information was used to set up a method to screen for presence of the CYP2C41 gene in a larger cohort of canine subjects. Primer pairs specific for exon 3 of CYP2C21 (EX3-F/R) and exon 7 of CYP2C41 (EX7-F/R) were used for single gene amplification, as well as for multiplex PCR as shown in Figure 2. PCR amplification was performed in a real-time PCR cycler, and the use of SYBR green dye allowed the monitoring of amplification of double-stranded DNA. Subsequent melting curve analysis revealed Tm values of 78.8°C and 83.6°C for the CYP2C21 and CYP2C41 amplicons, respectively (Figure 2A). So, both amplicons could be separately identified using the multiplex PCR approach. Representative PCR amplicons were then subjected to agarose gel electrophoresis, and this analysis confirmed the correct sizes of the amplicons for CYP2C41 exon 7 (primers EX7-F/R, 182 bp) and CYP2C21 exon 3 (primers EX3-F/R, 110 bp). However, a limitation of this method is that discrimination between the homo- or heterozygous occurrence of the CYP2C41 gene is not possible (Figure 2B).

### Determination of CYP2C41 Recombination Sites

Genomic sequences only recently became available from two CYP2C41 positive Labrador Retriever and Basenji subjects (see Table 2). This enabled the complete assembly of the canine CYP2C gene cluster as presented in Figure 1C. Of note, the site of CYP2C41 gene integration into the canine genome (site of recombination, SOR) is characterized by a specific sequence (5′-GCGGTTTAGCGCC-3′) that is duplicated upstream and downstream of the CYP2C41 gene and represents the break point of the CYP2C41 gene integration (see Figure 1C). PCR amplifications across recombination sites for a larger number of CYP2C41 positive (SOR1 and SOR2) and CYP2C41-negative (SOR) subjects revealed that the sequences on either side of this break point were highly conserved and even the gray wolf showed homologous sequences flanking SOR1 and SOR2 (Supplementary Figure 1). Only in very few exceptions, namely few Bulldog, Shar-Pei, and Kangal dogs, additional sequence insertions were detected upstream from SOR1 (Supplementary Figure 2).

### PCR-Based Method for CYP2C41 Genotyping

**Figure 3:**
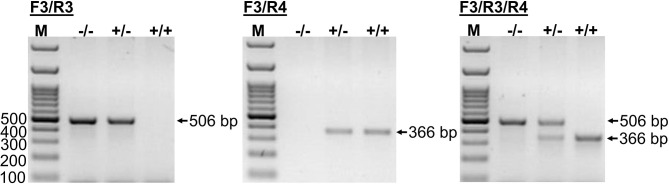
CYP2C41 genotyping. PCR for simultaneous detection of SOR (primers SOR-F3/R3) and/or SOR1 (primers SOR1-F3/R4) allows exact CYP2C41 genotyping for all three genotypes: (+/+), homozygous positive; (+/–), heterozygous; and (–/–), homozygous negative. The PCR products were separated on 2% agarose gels followed by visualization with ethidium bromide. Representative data for all three CYP2C41 genotypes are indicated. Amplification with SOR-F3/R3 (506 bp fragment) indicated the absence of the CYP2C41 gene, whereas amplification with SOR1-F3/R4 (366 bp fragment) indicated the presence of the CYP2C41 gene. Accordingly, CYP2C41(-/-) subjects only revealed the SOR-F3/R3 506 bp fragment, whereas CYP2C41(+/+) subjects only showed the SOR-F3/R4 366 bp fragment, and heterozygous CYP2C41(+/-) dogs were positive for both fragments (366 bp + 506 bp). M, molecular weight DNA marker.

This novel genomic information enabled the setting up of an additional PCR method that allowed the exact determination of the CYP2C41 genotype of a subject from a genomic DNA sample. In this approach, amplification with primers F3/R4 flanking SOR1 resulted in the amplification of a 366 bp fragment, demonstrating the presence of the CYP2C41 gene. In contrast, amplification with primers F3/R3 flanking SOR amplified a 506 bp PCR fragment that indicated the absence of the CYP2C41 gene. Combination of the forward primer F3 with both reverse primers R3 and R4 allowed the detection of the heterozygous CYP2C41(+/–) genotype by amplification of both (366 bp + 506 bp) fragments. In this assay, exclusive occurrence of the 366 bp or the 506 bp fragment indicated the homozygous CYP2C41(+/+) or CYP2C41(–/–) genotypes, respectively (Figure 3).

### CYP2C41 Genotyping of 1,089 Subjects From 36 Dog Breeds

Using this genotyping method, a cohort of 1,089 subject from 36 different canine breeds was genotyped for the occurrence of the CYP2C41 gene. As indicated in Table 5, the breeds Bearded Collie, Bernese Mountain Dog, Boxer, Briard, French Bulldog, and Irish Wolfhound revealed the most striking results, as among the 29–30 subjects from each tested breed, no subject was CYP2C41 positive. On the contrary, Chinese Char-Pei, Siberian Husky, Schapendoes and Kangal subjects showed relatively high CYP2C41 allelic frequencies of 67, 57, 43, and 34%, respectively, while the results from the other dog breeds fell somewhere in between these values. Of note, subjects with CYP2C41 genes on both alleles [CYP2C41(+/+)] were quite rare in most of the breeds (Table 5).

**Table 5:** CYP2C41 genotyping of 1,089 subjects from 36 dog breeds.

		**CYP2C41 genotype**			
**Dog Breed**	***N***	**-/-**	**+/-**	**+/+**	**CYP2C41 allele frequency (%)**
Bearded Collie	30	30	0	0	0
Bernese Mountain Dog	30	30	0	0	0
Boxer	30	30	0	0	0
Briard	29	29	0	0	0
French Bulldog	30	30	0	0	0
Irish Wolfhound	30	30	0	0	0
Australian Shepherd	30	29	1	0	1.7
Old English Sheepdog	30	29	1	0	1.7
Golden Retriever	30	29	1	0	1.7
Labrador Retriever	30	29	1	0	1.7
Bulldog	32	30	2	0	3.1
Australian Cattle Dog	30	28	2	0	3.3
Border Collie	30	28	2	0	3.3
Collie	30	28	2	0	3.3
Dobermann Pinscher	30	28	2	0	3.3
Greyhound	30	28	2	0	3.3
Waeller	30	28	2	0	3.3
Whippet	30	28	1	1	5.0
German shepherd dog	31	28	3	0	4.8
Belgian Malenois	30	27	1	2	8.3
Pug Dog	30	27	3	0	5.0
Berger Blanc Suisse	30	27	3	0	5.0
Nova Scotia Duck Tolling Retriever	30	26	3	1	8.3
Magyar Vizsla	29	24	5	0	8.6
Beagle	34	28	6	0	8.8
Jack Russel Terrier	30	24	6	0	10.0
Shetland Sheepdog	30	24	5	1	11.7
Chihuahua	27	21	5	1	13.0
Elo	30	22	7	1	15.0
Kelpie	30	20	10	0	16.7
Borsoi	30	20	10	0	16.7
Groenendael	31	19	11	1	21.0
Kangal	35	17	12	6	34.3
Schapendoes	30	9	16	5	43.3
Siberian Husky	29	5	15	9	56.9
Chinese Shar-Pei	32	2	17	13	67.2

*CYP2C41 genotypes: (–/–), genotype with two mutant alleles, homozygous for the deletion; (+/–), genotype with one CYP2C41 and one mutant allele, heterozygous for the deletion; (+/+), genotype with two CYP2C41 alleles, homozygous; N, number of dogs studied for each breed*.

### Breed-Clustering of the CYP2C41 Polymorphism in Comparison With MDR1 and CYP1A2 Polymorphisms

**Figure 4:**
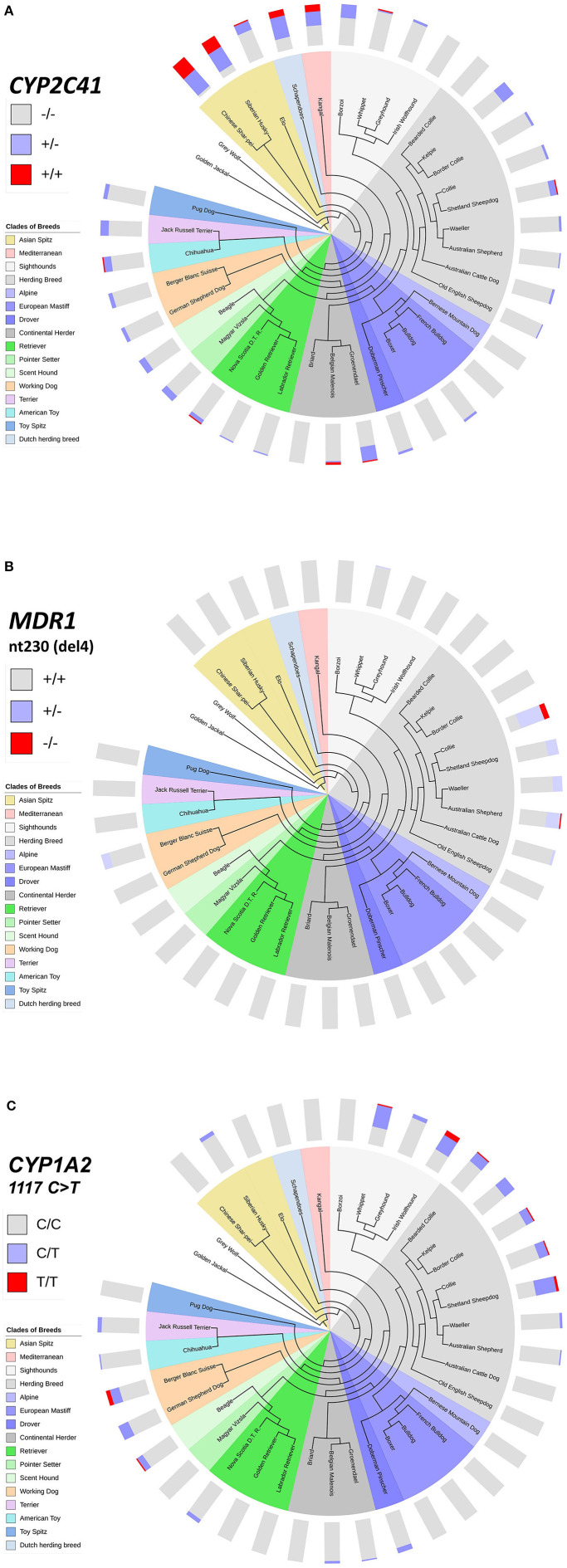
Comparative genotyping for CYP2C41, MDR1, and CYP1A2. All subjects listed in Table 5 were genotyped for CYP2C41 **(A)**, and also for the occurrence of the nt230(del4) MDR1 mutation **(B)**, and the CYP1A2 1117C>T single nucleotide polymorphism **(C)**. Charting of phylogenetic relationships and rooting of the 36 analyzed breeds was carried out according to Parker et al. (20). The phylogenetic tree was adapted to and displayed with the Interactive Tree Of Life (iTOL) tool (http://itol.embl.de/). The clustering of breeds (see color code) was carried out according to Parker et al. (20). The breeds Schapendoes, Elo, and Groenendael were included and grouped according to their breed history. Bar charts show color-coded percentage genotype distributions.

This inhomogeneous distribution across breeds was not surprising, as similar results had been detected before for other pharmacogenetic markers, such as the nt230(del4) MDR1 mutation (14, 17–19), or the CYP1A2 1117C>T transition (7, 16). To analyze if there was any overlap in the breed distribution pattern between these three pharmacogenetic markers, all 1,089 subjects were additionally genotyped for the CYP1A2 1117C>T marker. The nt230(del4) MDR1 genotypes were already known for each subject based on data from the institute‘s pharmacogenetic diagnostic service. Genotyping data were then plotted on cladograms that were adapted from Parker et al. (20). The CYP1A2 1117C>T polymorphism most frequently occurred in sighthounds, herding breeds and working dogs, but was also detected in some other clades (Figure 4C), while the nt230(del4) MDR1 mutation occurred exclusively in Whippets, herding breeds and working dogs (Figure 4B). In contrast, the CYP2C41 gene occurred in all breed clades (Figure 4A), with most frequent occurrence at the more ancient Asian Spitz clade, as well as Schapendoes and Kangal breeds. Based on this analysis, the CYP2C41 polymorphism can be regarded as the most widespread among the three markers tested.

## Discussion

### The CYP2C Family in Different Species

Among all CYPs involved in hepatic drug metabolism, the subfamily CYP2C is of particular interest in the context of canine-to-human comparison, as notable species differences exist in the CYP2C subfamily. At present, four CYP2C genes have been identified in humans (21), five in rats (22), 15 in mice (23), two in cats (24), and two in horses (25). Genetic polymorphisms in CYP2C genes have been shown to be of clinical relevance in humans (26). As an example, CYP2C9^*^2 or ^*^3 allele carriers are poor metabolizers of a number of clinically used medications, and have increased risk for adverse drug reactions, such as can occur after the administration of tolbutamide, NSAIDs, or warfarin (27). Although the clinical significance of canine CYP2C41 gene deletion polymorphism is not entirely clear, it can be stated that a gene deletion polymorphism within the CYP2C gene cluster is unique in canines.

### Current Characterization of Canine CYP2C41

Blaisdell et al. first analyzed the canine CYP2C family more in detail (13). They screened a canine liver cDNA library for closely homologous sequences with a 275 bp probe derived from CYP2C21. They identified a new canine CYP2C cDNA sequence of 1.800 bp with a complete open reading frame, encoding for the 489 aa CYP2C41 protein. Canine CYP2C21 and CYP2C41 have 70% nucleotide and amino acid sequence identities. Of note, CYP2C41 is more homologous to human CYP2Cs (amino acid sequence similarity ~75%) than CYP2C21 (amino acid sequence similarity ~ 70%). By using PCR-based testing, it was shown that from 28 individual subjects (18 Beagles and 10 mixed breeds) all were CYP2C21 positive, but only 4 (14%) were positive for CYP2C41. As these CYP2C41-positive subjects included Beagles, mixed breeds, and male and female subjects, the presence of the CYP21C41 gene was considered to be independent of the breed and gender of the subject (13). Subsequent studies then focused on the catalytic activity of canine CYP2C proteins and addressed the question of if differences in the catalytic activity between human and canine liver microsomes can be attributed to canine genetic variability including CYP2C41. In this context, Graham et al. analyzed the expression of canine liver CYPs and detected CYP2C41 mRNA in only 5 out of 11 subjects (28). In a more recent study, Martinez et al. used TaqMan gene expression assays to quantitatively compare the gene copy numbers for CYP2C41 and UGT1A (29). They were able to discriminate potential heterozygous and homozygous carriers of the CYP2C41 gene. In addition, they analyzed the absolute expression of 11 drug-metabolizing CYPs by means of LC-MS/MS, including CYP2C41, from liver samples of 59 subjects from different canine breeds. They found quantifiable amounts of CYP2C41 only in 12 of the 59 samples. Compared to other CYPs such as CYP2D15 or CYP3A12, CYP2C41 showed relatively low protein abundance in the liver (29). However, 11 of these 12 samples seemed to only contain one copy of the CYP2C41 gene, which could have led to an underestimation of the relative protein abundance of CYP2C41 in the canine liver.

### Catalytic Activity of CYP2C41

Whereas in humans CYP2C8, CYP2C9, and CYP2C19 significantly contribute to liver drug metabolism (these enzymes account for ~5, 13, and 7% of the metabolism of clinically used drugs, respectively) (27), the level of liver metabolism is not yet clear for the canine CYP2C21 and CYP2C41 enzymes. In canines, both proteins constitute ~24% of the total hepatic CYP proteins (30). In general, little is known about the catalytic activity and substrate specificity of CYP2C21 and CYP2C41, and so the clinical importance of the CYP2C41 gene deletion polymorphism remains largely unclear. Nevertheless, diclofenac and S-mephenytoin (marker substrates for human CYP2C9 and CYP2C19, respectively), as well as tramadol have already been identified as CYP2C21/CYP2C41 substrates *in vitro* (31–33), indicating potential pharmacogenetic relevance of these genes. However, a limitation of the present study is that the catalytic activity of CYP2C41 was not analyzed.

### CYP2C41 Gene Deletion Polymorphism

There are two possible explanations of how the CYP2C41 gene polymorphism developed. The first possibility is that canine CYP2C21 and CYPC41 originated from a gene duplication event in the same gene cluster, and CYP2C41 was later deleted in certain breed lines by recombination. In this scenario, the CYP2C41 gene variation can be classified as a gene deletion polymorphism. The second possibility could just be a polymorphic gene duplication event in the CYP2C gene cluster that only occurred in individual subjects from certain breed lines. Although the second possibility cannot be completely ruled out, a gene deletion event seems much more likely for several reasons. First, CYP2C41 has much higher sequence identity with human CYP2C genes and proteins than CYP2C21. Second, the CYP2C41 gene is somewhat smaller than the CYP2C21 gene, making gene duplication from CYP2C21 unlikely. Third, the recently available gray wolf genome reveals two genes, CYP2C21 and CYP2C41, within the CYP2C cluster and both are highly homologous to the canine orthologs. This overall provides support for speculation that CYP2C41 was the original gene in this gene cluster, and that after the duplication and divergence of the CYP2C21 gene, CYP2C41 was deleted by recombination. Finally, as highly homologous sequences upstream and downstream of the CYP2C41 gene were found, a deletion at these sites by homologous recombination seems likely, but an insertion seems unlikely. Based on these points, it can also be speculated that this deletion event occurred independently several times in individual subjects from different dog breed lines. However, as clustering of this CYP2C41 gene variation was found in certain breeds, the CYP2C41 gene deletion event might also have occurred only once in a single individual subject. Across all types of genetic variations, polymorphic deletions of entire genes are quite unusual. However, in humans a partial CYP2D6 gene deletion (34), and a complete deletion of the CYP2A6 gene (35) have been described.

## Conclusion

In the present study an exact characterization of the canine CYP2C41 gene deletion polymorphism at the genomic level is provided. In addition, a genotyping method that was already tested in 1,089 individual subjects from 36 different breeds is presented. Interestingly, the site of gene deletion was identical for all CYP2C41 negative subjects, and all CYP2C41 subjects showed highly homologous sequence domains upstream and downstream from the CYP2C41 gene, at which homologous recombination likely occurred. The CYP2C41 genotyping test can now be used in future canine pharmacokinetic studies to identify genetically based poor or extensive drug metabolizers. These results, together with more extensive *in vitro* drug screening for CYP2C41 substrates, will help to determine the clinical relevance of CYP2C41, and to optimize drug treatment. Although the CYP2C41 relative protein abundance in the canine liver seems not to be very high, this CYP could substantially contribute to hepatic drug metabolism in subjects that express CYP2C41 at both alleles, and when CYP2C41 shows higher catalytic activity in response to a given drug than other hepatic metabolic enzymes.

## Data Availability Statement

The datasets presented in this study can be found in online repositories. The names of the repository/repositories and accession number(s) can be found in the article/supplementary material.

## Ethics Statement

Ethical review and approval was not required for the animal study because blood samples from client-owned dogs were originally nt230(del4) MDR1 genotyped for clinical diagnostic reasons as part of the pharmacogenetic diagnostic service at the host institute. Some of these DNA samples then were used for retrospective CYP2C41 genotyping. The dog owners gave consent that the DNA samples can be used for subsequent scientific studies instead of being discarded. No samples were taken specifically for the retrospective analysis presented in the current manuscript and, therefore, this re-analysis did not require ethical approval.

## Author Contributions

EK, SL, and JG conceived the experiments, analyzed and interpreted the results. EK, SL, and CP performed the experiments. EK and JG wrote the manuscript. All authors approved the manuscript.

## Conflict of Interest

The authors declare that the research was conducted in the absence of any commercial or financial relationships that could be construed as a potential conflict of interest. The authors declare that nt230(del4) MDR1 genotyping is a commercial diagnostic service of their host institute.
