# Supplementary material for: Sequencing of the Canine Cytochrome P450 CYP2C41 Gene and Genotyping of Its Polymorphic Occurrence in 36 Dog Breeds
Source: Front Vet Sci. 2021 Apr 22;8:663175. doi: 10.3389/fvets.2021.663175 (PMC8100205; doi:10.3389/fvets.2021.663175)
Supplement: Supplementary file 1 [file Data_Sheet_1.pdf]

Sequencing of the Canine Cytochrome P450 CYP2C41 Gene and Genotyping of Its Polymorphic Occurrence in 36 Dog Breeds

Emre Karakus†, Clarissa Prinzinger†, Silke Leiting, Joachim Geyer*

Institute of Pharmacology and Toxicology, Faculty of Veterinary Medicine, Justus Liebig University Giessen, Schubertstr. 81, 35392 Giessen, Giessen, Germany

†These authors contributed equally to this work and share first authorship

*Correspondence to Joachim.M.Geyer@vetmed.uni-giessen.de

Supplementary Figures 1 and 2

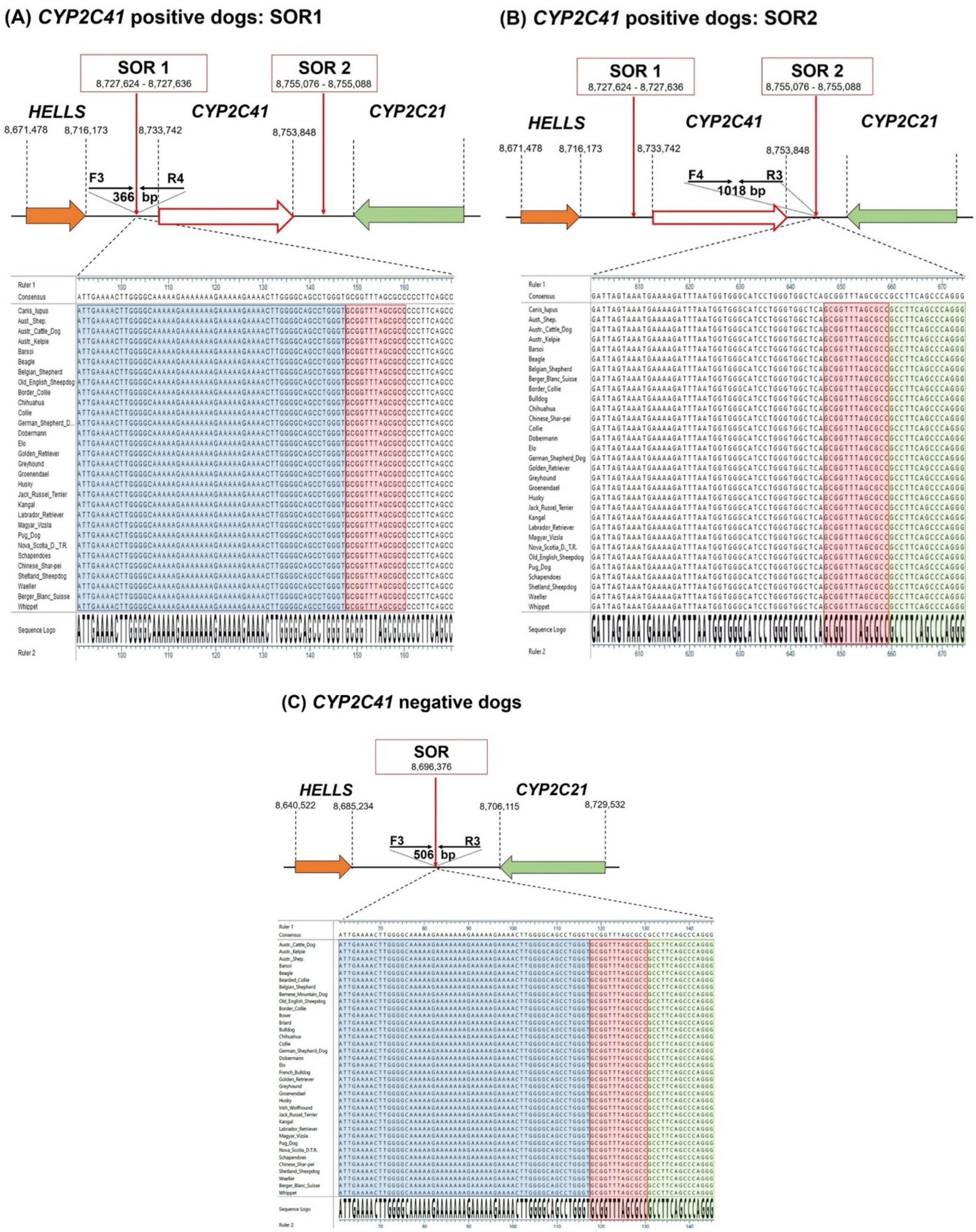

Supplementary Figure 1

Multiple sequence alignments at the sites of recombination, being SOR1 **(A)** and SOR2 **(B)** of CYP2C41 positive dogs as well as SOR **(C)** of CYP2C41 negative dogs. Respective PCR fragments were amplified with the primer combinations F3/R4 (SOR 1), F4/R3 (SOR 2), or F3/R3 (SOR) and subjected to DNA sequencing. Individual sequencing data were aligned with the MegAlign tool of the DNASTAR software. Conserved sequences at the break points are marked in red (see also **Figure 1C**) and the upstream and downstream regions were highlighted in blue and green, respectively. In addition, the exact positions of the respective gene segments are indicated. The sequence from a CYP2C41 positive gray wolf (*Canis lupus,* GenBank sequence HG994413.1) was included for comparison.

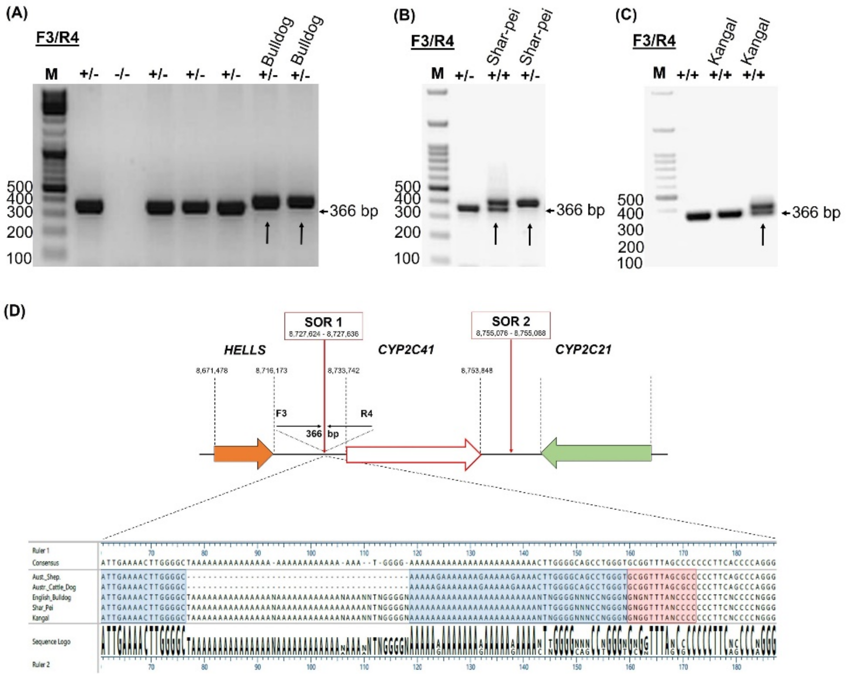

Supplementary Figure 2

Sequence variation of individual CYP2C41 positive dogs upstream of the site of recombination 1 (SOR1). The CYP2C41 genotype is indicated as follows: (−/−), genotype with two mutant alleles, homozygous for the CYP2C41 deletion; (+/−), genotype with one CYP2C41 allele and one mutant allele, heterozygous for the CYP2C41 deletion; (+/+), genotype with two copies of the CYP2C41 gene. PCR fragments were amplified with the primer combination F3/R4 and visualized on 2% agarose gels. Band shifts indicate sequence variations of individual Bulldog **(A)**, Shar-Pei **(B)**, and Kangal dogs **(C)**. M, molecular weight marker. **(D)** After DNA sequencing, individual sequences were aligned with the MegAlign tool of the DNASTAR software. Conserved sequences at SOR 1 are marked in red (see also **Supplementary Figure 1A**). Individual dogs revealed sequence insertions upstream of the break point of SOR1.
